# Plasmon-Emitter Hybrid Nanostructures of Gold Nanorod-Quantum Dots with Regulated Energy Transfer as a Universal Nano-Sensor for One-Step Biomarker Detection

**DOI:** 10.3390/nano10030444

**Published:** 2020-03-01

**Authors:** Xuemeng Li, Yingshuting Wang, Quanying Fu, Yangyang Wang, Dongxu Ma, Bin Zhou, Jianhua Zhou

**Affiliations:** 1 Key Laboratory of Sensing Technology and Biomedical Instruments of Guangdong Province, School of Biomedical Engineering, Sun Yat-sen University, Guangzhou 510275, China; lixuemeng6758@126.com (X.L.); wangysht@mail2.sysu.edu.cn (Y.W.); fuqy3@mail2.sysu.edu.cn (Q.F.); wang45yangyang@163.com (Y.W.); ma023ma@126.com (D.M.); zhoub56@mail2.sysu.edu.cn (B.Z.); 2 Division of Engineering in Medicine, Department of Medicine, Brigham and Women’s Hospital, Harvard Medical School, Cambridge, MA 02139, USA

**Keywords:** plasmonic-emitter hybrid nanostructure, localized surface plasmon resonance, fluorescence resonance energy transfer, gold nanorod, quantum dot, biomarker

## Abstract

Recently, biosensing based on weak coupling in plasmon-emitter hybrid nanostructures exhibits the merits of simplicity and high sensitivity, and attracts increasing attention as an emerging nano-sensor. In this study, we propose an innovative plasmon-regulated fluorescence resonance energy transfer (plasmon-regulated FRET) sensing strategy based on a plasmon-emitter hybrid nanostructure of gold nanorod-quantum dots (Au NR-QDs) by partially modifying QDs onto the surfaces of Au NRs. The Au NR-QDs showed good sensitivity and reversibility against refractive index change. We successfully employed the Au NR-QDs to fabricate nano-sensors for detecting a cancer biomarker of alpha fetoprotein with a limit of detection of 0.30 ng/mL, which displays that the sensitivity of the Au NR-QDs nano-sensor was effectively improved compared with the Au NRs based plasmonic sensing. Additionally, to demonstrate the universality of the plasmon-regulated FRET sensing strategy, another plasmon-emitter hybrid nano-sensor of Au nano-prism-quantum dots (Au NP-QDs) were constructed and applied for detecting a myocardial infarction biomarker of cardiac troponin I. It was first reported that the change of absorption spectra of plasmonic structure in a plasmon-emitter hybrid nanostructure was employed for analytes detection. The plasmon-regulated FRET sensing strategy described herein has potential utility to develop general sensing platforms for chemical and biological analysis.

## 1. Introduction

Plasmonic nanostructures generate extremely confined and enhanced electromagnetic fields as well as display localized surface plasmon resonance (LSPR) peaks in absorption or scattering spectra under the incident light [1,2,3,4]. LSPR peaks of plasmonic nanostructures are sensitive to small changes of the adjacent refractive index (RI) caused by the molecular binding events on the metal/dielectric interfaces [5,6,7]. Therefore, plasmonic nanostructures have lately attracted considerable attention as a new class of simple, rapid, and label-free nano-sensors, which enable many important applications, such as biomarkers detection, environmental pollution analysis, and food quality monitoring [8,9,10,11,12,13]. To exploit new sensing schemes and improve the sensitivity of plasmonic nano-sensors [14,15], efforts have been devoted to the plasmonic hybrid nanostructures, such as plasmon-exciton hybrid nanostructures [16,17], plasmon-dielectric hybrid nanostructures [18], and plasmon-emitter hybrid nanostructures [19,20]. Among them, plasmon-emitter hybrid nanostructures exhibit the advantages of simplicity and high sensitivity, and are widely concerned [21,22].

**Scheme 1:**
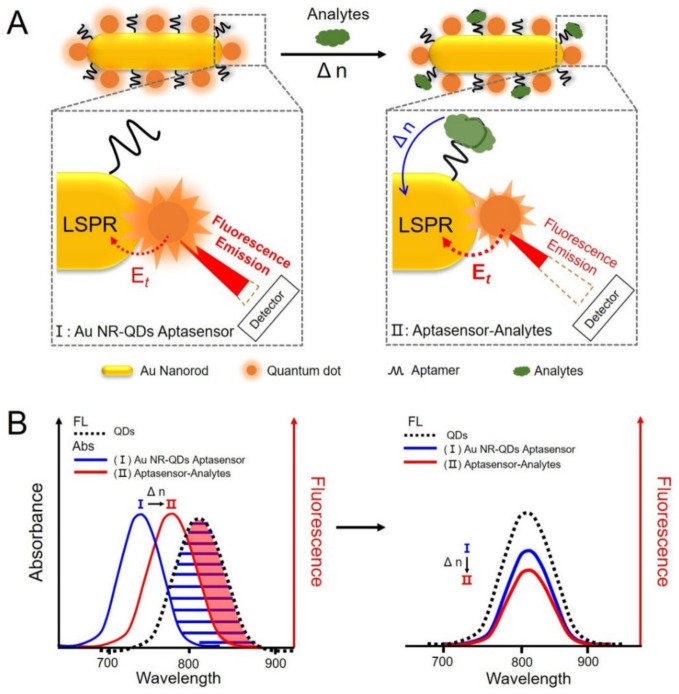
Schematic illustration of a plasmon-regulated fluorescence resonance energy transfer (plasmon-regulated FRET) sensing system based on the Au nanorod-CdSeTe quantum dots (Au NR-QDs) apta-sensor. (**A**) The energy transfer (*E_t_*) of the Au NR-QDs apta-sensor before (I) and after (II) capturing analytes. (**B**) The changes of absorption and fluorescence emission spectra in the same process of (**A**).

In a plasmon-emitter hybrid nanostructure, the emitter generates fluorescence under the excitation of light, and the plasmonic nanostructure is introduced to regulate the fluorescence intensity due to the weak coupling between the plasmonic nanostructure and the emitter [23,24]. This coupling degree in a plasmon-emitter hybrid nanostructure could be changed not only by the distance between the plasmon and emitter, but also by the degree of spectral overlap between the absorption spectrum of the plasmonic nanostructure and the emission spectrum of the emitter [25,26,27]. These changes of the coupling degree that depended on the distance or spectral overlap make it easy to achieve biochemical sensing by associating the molecular specific recognition with the previously mentioned properties of plasmon-emitter hybrid nanostructures. To date, the coupling mechanisms of plasmon-emitter hybrid nanostructures, known as nano-sensors, are employed for sensitive and rapid detection of metal ions [28], small molecules [29,30,31], proteins [32,33], and pathogens [34,35], which have been mainly classified into two types. One is based on plasmon-enhanced fluorescence (PEF) in which the confined electromagnetic fields of the plasmonic nanostructure enhance the fluorescence intensity of the emitter [14,36]. The recognition of analytes is designed to enhance the coupling by changing the plasmon-emitter distance, and the fluorescence intensity is further increased. Those nano-sensors possess the advantages of a simple structure and high sensitivity but are confronted with the limitations of compromised reproducibility and time-consuming operations [25]. Another type of plasmon-emitter hybrid nano-sensor relies on fluorescence resonance energy transfer (FRET) in which a non-radiative energy transfer (*E_t_*) from the emitter to the plasmonic nanoparticle is led by the LSPR of the plasmonic nanoparticle [15,37,38,39]. The recognition of analytes can alter the plasmon-emitter distance or destroy plasmon-emitter hybrid nanostructures, which causes a variation of fluorescence intensity. Those nano-sensors usually work in solutions with the merits of highly sensitive and the convenience of washing free [21]. However, they still suffer from some shortcomings, such as the analytes-dependent design of the sensing system, the complexity of sensor fabrication, and the lack of universality. Therefore, it is still desired to propose novel sensing strategies that benefit the superiorities of nano-photonics to develop biosensors with the merits of easy fabrication, high sensitivity, and certain universality. Herein, we proposed a new sensing strategy based on FRET, defined as plasmon-regulated FRET strategy, which is demonstrated by a plasmon-emitter hybrid nano-sensor based on gold nanorod-quantum dots (Au NR-QDs). As shown in Scheme 1, the plasmon-emitter hybrid nanostructures of Au NR-QDs were prepared by partially linking QDs onto the surface of Au NRs through covalent bonds. The energy of the excited QDs (i.e., emitter) is partly transferred to Au NRs (i.e., plasmon) through a non-radiative FRET process [29], and the remaining radiative energy supports the Au NR-QDs apta-sensor to emit fluorescence. The recognition of molecules by the aptamer could alter the RI (*Δn*) surrounding the Au NR-QDs apta-sensor, which induces the shift of the LSPR peak of Au NR and results in the increase of spectral overlap between the absorption spectrum of Au NR and the emission spectrum of QDs. Then, the regulation of *E_t_* is triggered and further leads to the variation of fluorescence intensity for biosensing. Distinguishing from the previously mentioned plasmon-emitter hybrid nano-sensors based on PEF or FRET, our plasmon-regulated FRET nano-sensors have the constant plasmon-emitter distance, and apply the absorption spectrum of the plasmonic nanostructures to regulate the *E_t_* for biosensing. 

## 2. Materials and Methods 

### 2.1. Chemicals and Materials

Chloroauric acid hydrate (HAuCl_4_·3H_2_O, Lot#: F1719017), hexadecyltrimethylammonium bromide (CTAB, Lot#: E1719115), sodium borohydride (NaBH_4_), L-ascorbic acid (AA), silver nitrate (AgNO_3_), and cysteamine hydrochloride (Lot#: I1812063) were commercially available from Aladdin (Shanghai, China). Hydrochloric acid (HCl) was provided by the Guangzhou Chemical Reagent (Guangzhou, China). Carboxylic CdSeTe quantum dots (Lot#: 1977331) were purchased from ThermoFisher Scientific (Shanghai, China). N-(3-dimethylaminopropyl)-N-ethylcarbodiimide hydrochloride (EDC) and N-hydroxysuccinimide (NHS) were purchased from Sigma Aldrich (Shanghai, China). Glutaraldehyde (GA) was acquired from Damao Chemical Reagent (Tianjin, China). The amino-terminated AFP aptamer with the sequence of 5’-NH_2-_GTGACGCTCCTAACGCTGACTCAGGTGCAGTTCTCGACTCGGTCTTGATGTGGGTCCTGTCCGTCCGAACCAATC-3’ from a previous report was synthesized from BGI (Shenzhen, China) [40]. Human alpha fetoprotein (AFP, Lot#: L2C00201) was supplied by Shanghai Linc-Bio Science Co (Shanghai, China). AFP monoclonal antibody (Lot#: 2AFP-28) was obtained from Fapon Biotech Inc (Shenzhen, China). Human cardiac troponin I (cTnI, Lot#: ZKP13) and anti-cTnI monoclonal antibody (Lot#: XJ235) were all from Tongrenxin Biotechnology (Xiamen, China). 

### 2.2. Apparatus

The UV-vis absorption spectra were recorded by a UV-vis spectrophotometer (L3S, Inesa, Shanghai, China). The fluorescence emission spectra were obtained from a fluorescence spectrophotometer (FS5, Edinburgh Instruments Ltd., Livingston, UK). Transmission electron microscopy (TEM) images were captured by a transmission electron microscope (JEM-1400, JEOL Ltd., Tokyo, Japan) operated at 120 kV. Zeta potential measurements were performed on a zeta potential analyzer (ZS90, Malvern Instruments Ltd., Malvern, UK). Deionized water with a resistivity of 18.2 MΩ·cm was prepared through a Milli-Q Advantage A10 water system (Millipore, Billerica, MA, USA).

### 2.3. Synthesis of Au NRs

The Au NRs stabilized by CTAB were synthesized trough a seed-mediate method with some modification [41]. First, the 10 μL of HAuCl_4_·3H_2_O (0.125 M) was mixed with 4.7 mL of 0.1 M CTAB to obtain a homogenous solution under gentle stirring. Then, 0.3 mL of fresh-prepared ice-cold aqueous NaBH_4_ solution (0.01 M) was rapidly injected into the mixture. After stirring violently for 2 min, the brownish seed solution was obtained. Fresh seed solution was incubated in a water bath at 30 °C at about 3 h and then used for synthesizing Au NRs. 

For the synthesis of Au NRs, growth solution was prepared by adding 0.4 mL of 0.125 M HAuCl_4_·3H_2_O, 0.1 mL of 1 M HCl, and 0.12 mL of AgNO_3_ into 100 mL of 0.1 M CTAB solution under continuous gentle stirring. Next, 0.8 mL of 0.1 M AA was added, and the color of the mixture changed from orange to colorless. Lastly, 0.24 mL of seed solution was rapidly injected into the growth solution under vigorous stirring. After stirring for 5 min, the resultant solution was left undisturbed in a 30 ℃ water bath overnight to allow the complete growth of Au NRs.

### 2.4. Functionalization of Au NRs with Amino Groups

Amino-modified Au NRs were obtained from literature with some modification [42]. Briefly, as-prepared Au NRs solution was centrifuged at 9000× *g* for 30 min to remove the excess CTAB and ions in the growth solution. The precipitate was then re-dispersed in deionized water. Next, 0.1 mL of 50 mM cysteamine hydrochloride was added dropwise into 5 mL of Au NRs solution under sonication at 50 °C and kept for 0.5 h. After that, the solution was moved to a 50 °C water bath and kept incubated for 2 h to allow the complete modification of both sides and tips of the Au NRs. Lastly, as-prepared amino-modified Au NRs solution was centrifuged and washed by deionized water and stored at 4 °C for further use.

### 2.5. Preparation of Au NR-QDs Assemblies

A commercial carboxylic CdSeTe quantum dots (QDs) whose emission peak wavelength was ~800 nm was selected as the fluorescence emitter. Au NR-QDs assemblies were synthesized through an EDC/NHS method [43,44]. A total of 8 μM of QDs in borate buffer was diluted to 30 nM by phosphate buffer saline (PBS) buffer (0.01 M, pH 7.4). Then, 100 μL of 40 mM of EDC and NHS mixture was added into 5 mL of QDs solution to effectively activate the carboxylic groups on the surface of the QDs. After gently shaking for 2 h, 5 mL of as-prepared amino-modified Au NRs was added, and the mixture was shaken for 1 h. Lastly, the mixture was left undisturbed overnight under room temperature, and then centrifuged and washed by deionized water for later use.

### 2.6. Sensing Behavior of the Plasmon-regulated FRET Sensing System of Au NR-QDs

The sensing behavior of the plasmon-regulated FRET sensing system of Au NR-QDs was verified in an increasing RI environment. Au NR-QDs solutions were mixed with different glycerol/water mixtures. The RI conditions were set as 1.333, 1.336, 1.337, 1.339, 1.340, 1.341, 1.343, and 1.344, and the corresponding glycerol/water ratios were 0, 2.46%, 3.65%, 4.81%, 5.94%, 7.04%, 8.12%, and 9.18%, respectively. Three measurements were operated at each RI condition to determine the standard deviation (S.D.) of peak wavelength and fluorescence intensity.

### 2.7. Reversible RI-Dependent Properties of Au NR-QDs

The measurements of reversible RI-dependent properties of Au NR-QDs were performed as follows. First, Au NR-QDs solution was mixed with a certain amount of glycerol solution to obtain an RI value of 1.334 as the initial solution. The above Au NR-QDs solution was added with more glycerol solution to increase the RI value to 1.339, and then Au NR-QDs/glycerol solution with RI of 1.339 was added to the pure Au NR-QDs mixture to decrease the RI to 1.334. All of the above operations were repeated three times. The corresponding UV-Vis and fluorescence spectra were recorded after each operation.

### 2.8. Fabrication of Au NR-QDs Based Nano-sensors

The surfaces of Au NRs were modified with aptamers through a cross-linking method by GA [45]. First, 50 μL of GA solution (50 wt%) was added to 5 mL of Au NR-QDs solution and incubated for 30 min with gentle shaking. Then, the suspension was purified to get rid of the redundant GA, and re-dispersed in 2.5 mL of deionized water. Subsequently, 2.5 μL aptamers solution (100 μM) was dropped into the GA-functionalized Au NR-QDs mixture and incubated at room temperature for 2 h under gentle shaking. Antibodies (Ab) modified Au NR-QDs nano-sensor for cTnI detection was constructed by the following protocol. Furthermore, 2.5 μL cTnI antibody solution (2.273 mg/mL) was dropped into the 2.5 mL GA-functionalized Au NR-QDs mixture and incubated at room temperature for 2 h under gentle shaking.

### 2.9. Detection of AFP Using the Au NR-QDs Apta-sensor

For AFP detection, AFP stock solution was diluted into different concentrations by PBS, and those diluent AFP solutions were added into 600 μL of Au NR-QDs apta-sensor solution. The final concentrations of AFP were 0.1, 0.3, 1, 3, and 10 ng/mL, respectively. After each operation, solutions were incubated at room temperature for 30 min and the fluorescence spectra were carried out. The limit of detection (LOD) of the apta-sensor was calculated according to the 3*s_b_/m* criterion, where *m* is the slope of the first three experimental data and *s_b_* is the standard deviation of the blank (n = 3). The selectivity of the Au NR-QDs apta-sensor against other agents in serum was tested. As-prepared Au NR-QDs was incubated with PBS, bull serum albumin (BSA, 1 μg/mL), glucose (100 ng/mL), and AFP (10 ng/mL) at room temperature, respectively. After incubation for 30 min, the fluorescence spectra were recorded. 

### 2.10. Detection of AFP in Spiked Serum Samples

The AFP spiked serum samples were prepared by diluting AFP negative serum for 10-fold by PBS, and spiking with standard solution of AFP to obtain the final concentration from 0 to 6.4 ng/mL, respectively. All spiked serums were aged at room temperature for 10 min before use. Lastly, 10 μL of each spiked serum sample was incubated with 600 μL of Au NR-QDs apta-sensor solution for 30 min, and then the fluorescence spectra were recorded.

### 2.11. Detection of cTnI Using the Antibodies Modified Au NR-QDs and Au NP-QDs Nano-sensors

Antibodies modified Au NP-QDs nano-sensors were constructed by adding cTnI antibody (85 μL, 2.273 mg/mL) into 2.5 mL of GA-functionalized Au NP-QDs mixture. For cTnI detection, cTnI stock solution was diluted into different concentrations by PBS, and those diluent cTnI solutions were added into 600 μL of Au NR-QDs nano-sensor solution (the final concentrations of cTnI were 0.3, 1, 3, 10, and 15 ng/mL) or Au NP-QDs nano-sensor solution (the final concentrations of cTnI were 2, 4, 6, 8, and 10 ng/mL), respectively. At each operation, the solutions were incubated with sensors at room temperature for 30 min, and the corresponding fluorescence spectra were recorded, respectively.

## 3. Results and Discussion

### 3.1. Fabrication and Characterization of the Plasmon-Emitter Hybrid Nanostructures of Au NR-QDs Assemblies

**Figure 1:**
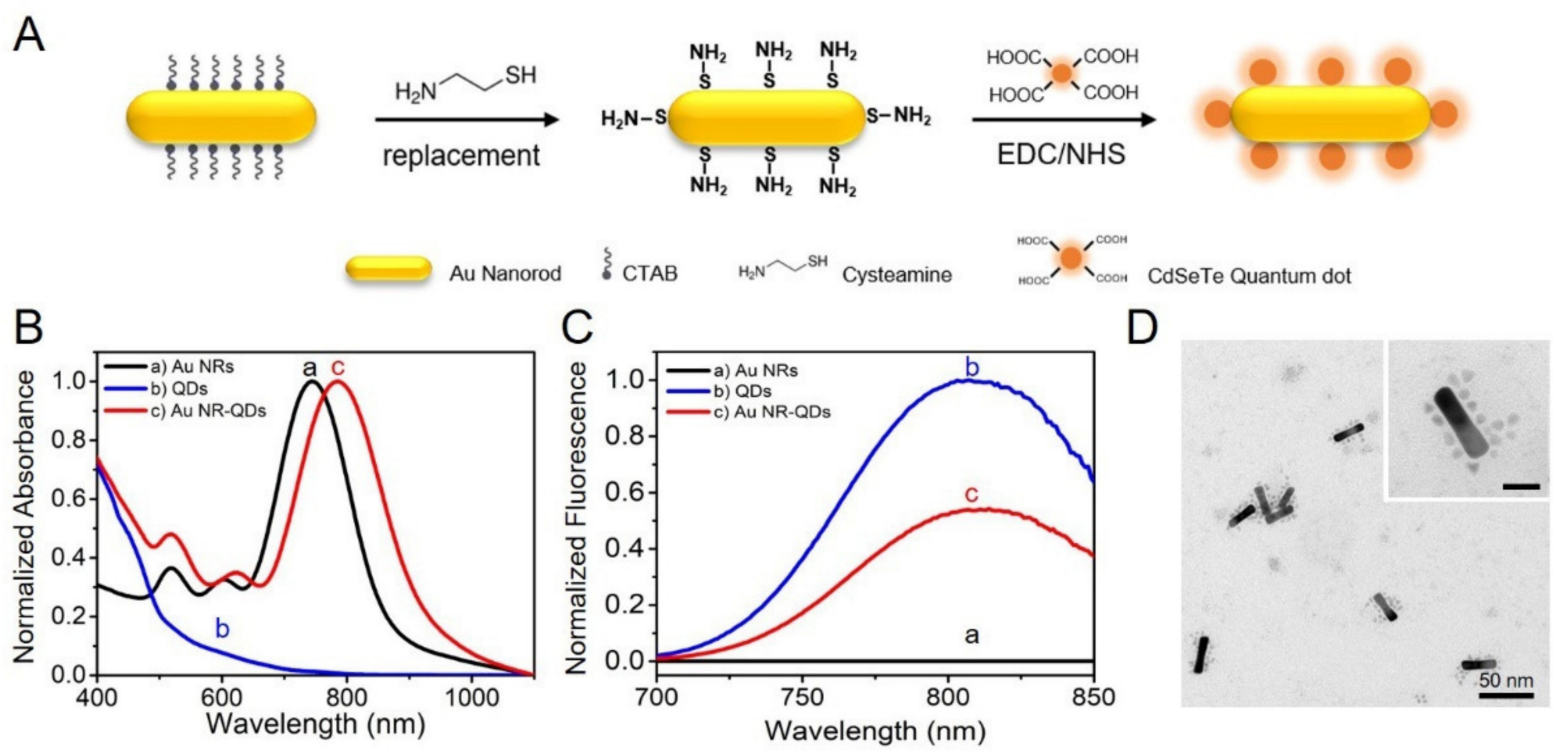
The fabrication and characterization of the plasmon-emitter hybrid nanostructures of Au NR-QDs assemblies. (**A**) The schematic illustration for the fabrication process of Au NR-QDs. (**B**) Normalized UV-vis absorption spectra of the Au NRs (a), QDs (b), and as-prepared Au NR-QDs (c). (**C**) Normalized fluorescence spectra of Au NRs (a), QDs (b), and Au NR-QDs (c) in the same process of (**B**), where the maximum fluorescence intensity of QDs was regarded as 1.0. (**D**) Transmission electron microscopy (TEM) images of Au NR-QDs. The inset is a single Au NR-QDs, and the scale bar is 25 nm.

Figure 1 shows that plasmon-emitter hybrid nanostructures of Au NR-QDs assemblies could be prepared with our synthesized Au NRs and commercial carboxyl-modified CdSeTe quantum dots (QDs). To fabricate Au NR-QDs assemblies, Au NRs with merits of easy preparation and adjustable absorption spectra are chosen as plasmonic nanostructures [46,47]. CdSeTe QDs with the narrow emission peak are selected as emitters [48]. The fabrication process of Au NR-QDs is illustrated in Figure 1A. First, Au NRs were synthesized by a seed-mediated growth method and then modified by cysteamine to obtain Au NRs-NH_2_, as previously reported [41,42], and the successful synthesis of Au NRs-NH_2_ was proven by a blueshift of LSPR peak (Figure S1) and a clear decrease of the zeta-potential from +35.3 mV to +21.2 mV after the modification of cysteamine. Then, Au NR-QDs were formed by covalently linking carboxyl-modified QDs onto the surface of Au NRs-NH_2_ with the participation of EDC/NHS. The interparticle distance between Au NRs and QDs is regarded as a constant due to the immobilization of covalent bonds.

The fabrication of Au NR-QDs was conducted by a series of experiments to ascertain optimal conditions (Figures S2–S5). Au NR-QDs assemblies were fabricated by covalently linking QDs (emission peak of 812 nm) with Au NRs (*λ*_LSPR_ of 744 nm), and the molar ratio of Au NRs: QDs was 1:130. Normalized absorption spectra of Au NRs (a), QDs (b), and Au NR-QDs (c) are shown in Figure 1B. After Au NRs were modified with QDs, a visible redshift of *λ*_LSPR_ of Au NRs from 744 nm to 786 nm was observed, while the absorption of QDs was negligible between 700 to 800 nm. It seems that the linkage of QDs evidently increased the surrounding RI near the surfaces of Au NRs and caused the redshift of *λ*_LSPR_ [49]. Figure 1C is the corresponding fluorescence spectra. QDs displayed a fluorescence emission peak at 812 nm under the excitation light of 450 nm. The fluorescence intensity was quenched dramatically (~45%) after linking Au NR with QDs, while Au NRs showed no fluorescent signal. It is reasonable that the fluorescence quenching of Au NR-QDs compared to QDs is induced by the occurrence of non-radiative *E_t_* from QDs to Au NRs [29,37]. Figure 1D presents the TEM images of Au NR-QDs. The aspect ratio of the Au NR-QDs based on the TEM images is calculated as ~2.1, with the length of 74 ± 2 nm and the diameter of 35 ± 2 nm. The surfaces of Au NRs were partially and dispersedly coated by 10 ± 2 of QDs with uniform distribution, which was direct evidence for the successful fabrication of Au NR-QDs. Moreover, the zeta-potentials declined from +21.2 mV of Au NRs-NH_2_ to −23.7 mV of Au NR-QDs, which can prove the successful fabrication of Au NR-QDs (Table S1). Taking the advantage of partial coverage of QDs on the surfaces of Au NRs, the residual surfaces of Au NRs can effectively sense the variation of surrounding RI and be further modified to fabricate RI-sensitive nano-sensors.

### 3.2. Sensing Behavior of the Plasmon-Regulated FRET Sensing System of Au NR-QDs

**Figure 2:**
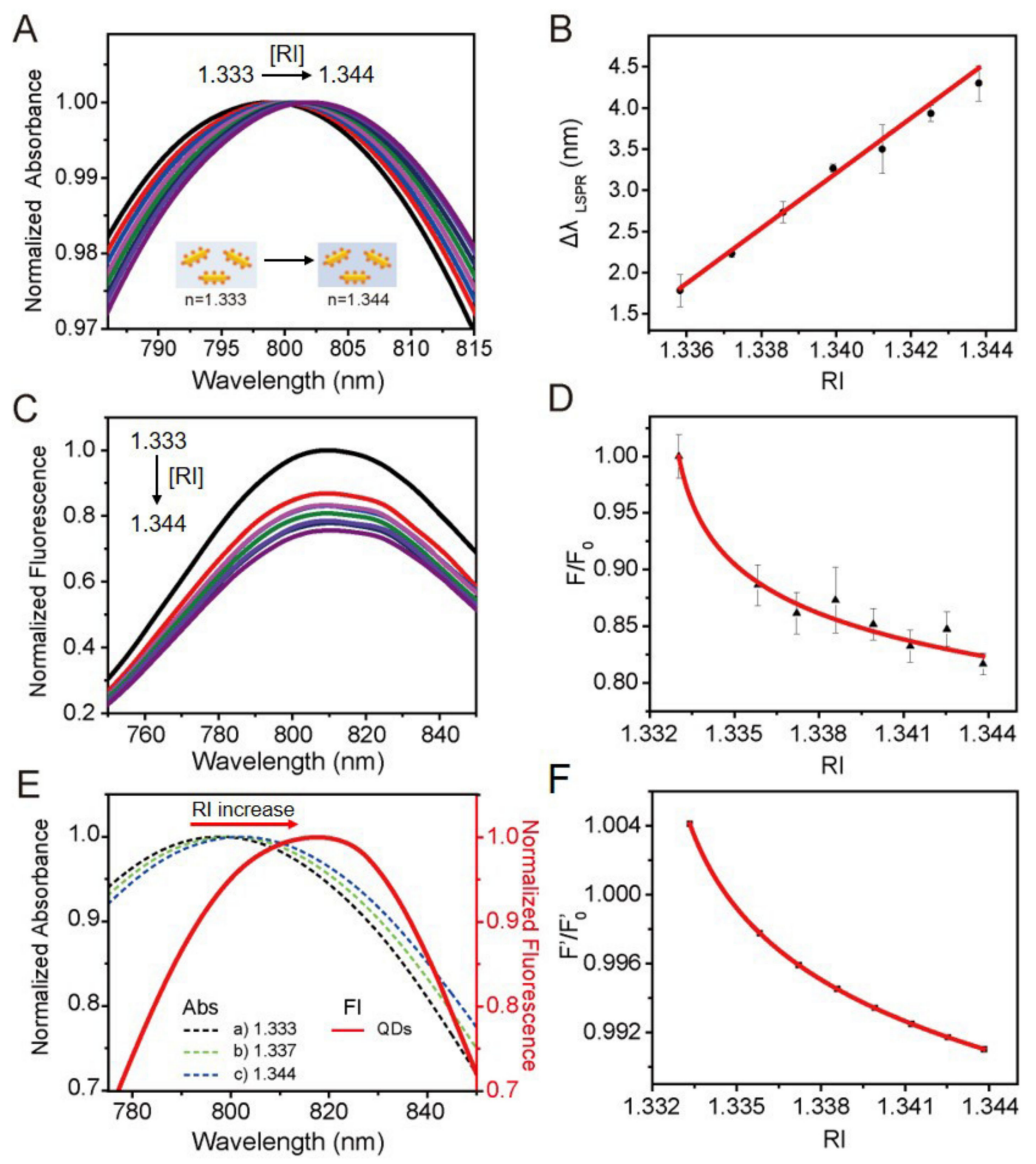
The response to refractive index (RI) for the plasmon-regulated FRET sensing system of Au NR-QDs. (**A**) Normalized UV-vis absorption spectra of Au NR-QDs in solutions with varying concentrations of water-glycerol mixtures with tuning environmental RI (*n* = 1.333, 1.336, 1.337, 1.339, 1.340, 1.341, 1.343 and 1.344), respectively. (**B**) The *Δλ*_LSPR_ for Au NR-QDs as a linear function of RI (*R*^2^ = 0.99) extracted from (**A**). (**C**) Normalized fluorescence spectra of Au NR-QDs in the same process as (**A**), where the maximum fluorescence intensity of Au NR-QDs when *n* = 1.333 was regarded as 1.0. (**D**) Relationship between the relative fluorescence intensity (*F/F*_0_) and environmental RI (*F/F_0_* = 0.624(*n* − 1.332)^−0.06^, *R*^2^ = 0.95). (**E**) Normalized absorption of Au NR-QDs with environmental RI of 1.333, 1.337, and 1.344, respectively, and the normalized fluorescence spectra of QDs. (**F**) Correlation between theoretical *F/F*_0_ (*F’/F’*_0_) and environmental RI.

Figure 2 demonstrates the RI sensitivity for the plasmon-regulated FRET sensing system of Au NR-QDs. Absorption and fluorescence spectra of Au NR-QDs in different water-glycerol mixtures (the environmental RI increased from 1.333 to 1.344) were recorded accordingly. The *λ*_LSPR_ of Au NR-QDs apparently red-shifted with the increase of RI (Figure 2A). A linear dependence was observed between the shift of *λ*_LSPR_ (Δ*λ*_LSPR_) and environmental RI (*R*^2^ = 0.99) in Figure 2B. The slope of the fitting line indicated that the RI sensitivity of Au NR-QDs was ~365 nm/RIU, which was equivalent to that of the bare Au NRs (~363 nm/RIU) in Figure S6. Fluorescence spectra and corresponding maximum fluorescence intensities against varied environmental RI were shown in Figure 2C,D, respectively. The fluorescence intensity of Au NR-QDs showed a decrease with increasing RI, and the change of fluorescence intensity was plotted as a power function of the RI (*F/F*_0_ = 0.624(*n* − 1.332)^−0.06^, *R*^2^ = 0.95), which was distinct from the tendency of *Δλ*_LSPR_ in Figure 2B. This power function suggests that our Au NR-QDs system holds higher sensitivity with smaller RI compared to the sensing based on the shift of *λ*_LSPR_. Figure S7 shows that the fluorescence intensity of QDs was relatively stable in varied RI, which indicates that the decrease of fluorescence intensity is mainly attributed to the effect of Au NRs rather than the fluorescent property of QDs themselves.

In principle, the decrease of fluorescence intensity of Au NR-QDs is due to the variation of *E_t_*. According to the FRET theory, the level of *E_t_* is positively related to the spectral overlap between the absorption of Au NRs and the emission of QDs. Then the relationship between spectral overlap (*S_OL_*) and relative fluorescence (*F/F*_0_) upon the change of RI (*n*) was further discussed, where *F*_0_ is the fluorescence intensity of the Au NR-QDs under an initial RI condition (see Supporting Information).

For a FRET system that holds a large level of *E_t_* efficiency, *F/F*_0_ can be calculated as follows:

(1) $$\frac{F}{F_{0}}=\frac{n^{4}}{AS_{OL}}$$

where A is a constant and $A=\frac{n_{0}{}^{4}}{S_{OL0}}$, where *n* is the RI of the medium and *S_OL_* expresses the spectral overlap integral between the normalized absorption of plasmonic structures and emission of emitters. Besides, *n*_0_ is the surrounding RI of the reference fluorescence intensity (*F*_0_), and *S*_*OL*0_ is the corresponding spectral overlap integral.

The normalized absorption spectra of Au NR-QDs in different RI conditions (*n*= 1.333, 1.337 and 1.344) and the normalized fluorescence emission spectrum of pure QDs were displayed in Figure 2E. With the RI increasing from 1.333 to 1.344, a clear increase of *S_OL_* can be observed along with the redshift of *λ*_LSPR_. A positive correlation between *S_OL_* and *n* was found (*S_OL_* = 85.83 (*n* − 1.332)^0.006^, *R*^2^ = 0.99), and S_OL0_ is calculated as 84.481 by the above function when n_0_ is set up as 1.333 (see Supporting Information). To calculate the theoretical *F/F*_0_ (i.e., *F’/F’*_0_) in the RI range from 1.333 to 1.344, the above function (*S_OL_* = 85.83(*n*−1.332)^0.006^) and the values of n_0_ and S_OL0_ were substituted into Equation (1). As shown in Figure 2F, the relationship between *F’/F’_0_* and RI can be approximately equal to *F’/F’_0_*=0.965(*n*-1.332)^-0.006^ (see Supporting Information), which is consistent with the change tendency of the experimental *F/F*_0_ in Figure 2D. The results confirm that the change of fluorescence intensity of the plasmon-regulated FRET sensing system is closely related to the variation of the LSPR properties of the plasmonic nanostructure, which is sensitive to the surrounding RI change [50]. Additionally, the value of the exponent for the change tendency of *F/F*_0_ is directly related to the RI sensitivity of LSPR property for the Au NR-QDs. Moreover, the Au NR-QDs exhibited good sensing reversibility and stability upon the change of RI between 1.333 and 1.339 for three cycles (Figure S8). Therefore, this plasmon-regulated FRET sensing system based on Au NR-QDs is capable of having a sensitive and reversible response to the environmental RI, which holds great potential for RI-based sensing of analytes.

### 3.3. Construction of the Au NR-QDs Nano-sensor

**Figure 3:**
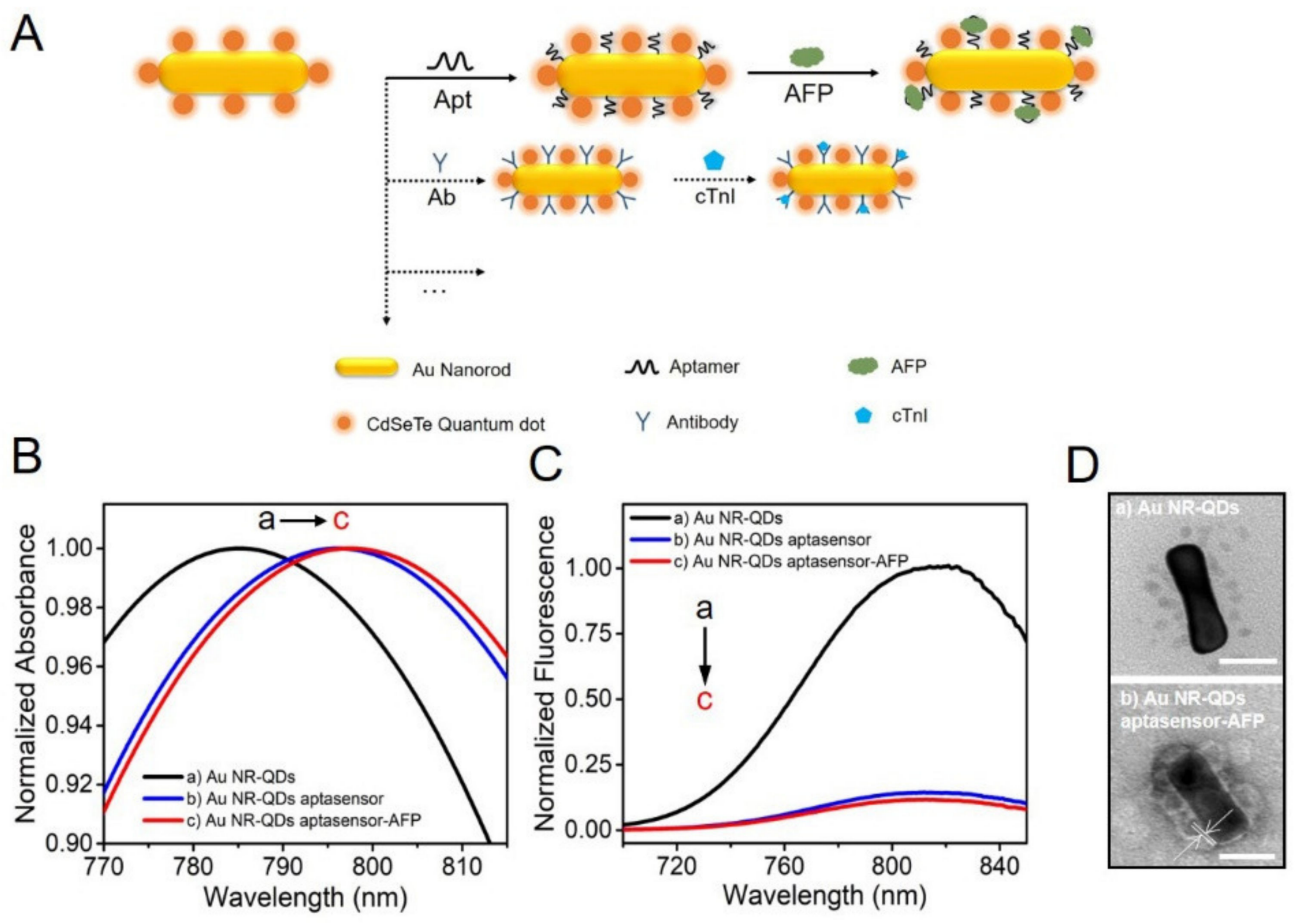
The Au NR-QDs apta-sensor for alpha fetal protein (AFP) detection. (**A**) Schematics for the construction of Au NR-QDs nano-sensors by modifying Au NR-QDs with different types of sensing layers such as aptamers and antibodies. (**B**) Normalized UV-vis absorption spectra and (**C**) normalized fluorescence spectra of Au NR-QDs (a), Au NR-QDs apta-sensor (b), and Au NR-QDs apta-sensor-AFP nanocomplex (c), where the maximum fluorescence intensity of Au NR-QDs was regarded as 1.00. (**D**) TEM images of Au NR-QDs (a) and Au NR-QDs apta-sensor-AFP nanocomplex (b), respectively. Scale bars are 25 nm.

To demonstrate the sensing ability of Au NR-QDs for analytes detection, an Au NR-QDs apta-sensor for AFP detection was constructed by modifying Au NR-QDs with aptamers by linking GA (Figure 3A). Aptamers are short and single-strand oligonucleotides that can tightly bind to a broad range of targets (e.g., proteins, drugs, metal ions), and have considerable advantages such as low cost, high affinity, good selectivity, easy labeling, and long-term chemical stability [51,52,53]. AFP is an important and commonly concerned biomarker for early diagnosis of cancers, especially for hepatocellular carcinoma (HCC) [35]. The concentration of AFP in healthy human plasma is usually less than 25 ng/mL and increases dramatically when HCC occurs [11]. As shown in Figure 3A, an Au NR-QDs apta-sensor can be constructed by immobilizing amino-terminated aptamers on the surfaces of Au NRs through the linkage of GA. The same modification protocol for various analytes detection can also be applied to other amino-terminated molecules such as antibodies.

The absorption and fluorescence spectra for the construction and application of Au NR-QDs apta-sensor were recorded in Figure 3B and Figure 3C, respectively. After the modification of aptamers, a visible redshift of *λ*_LSPR_ (10.9 nm) and a dramatic decline of fluorescence intensity (~85%) were observed. The zeta-potential declined from −23.7 ± 1.4 mV to −30.2 ± 3.2 mV after the modification of negative-charged aptamers on the surfaces of Au NR-QDs. All the above results confirmed that an Au NR-QDs apta-sensor was successfully obtained. Moreover, the negative-charged QDs and aptamers on the surfaces of Au NR-QDs would keep our Au NR-QDs apta-sensor stable and prevent the sensor from aggregation in the salt solution, which is important for sensing applications [54,55,56,57]. 

Then, the Au NR-QDs apta-sensor was applied to detect AFP of 20 ng/mL. After capturing AFP onto the Au NR-QDs apta-sensor, another redshift of *λ*_LSPR_ for 1.2 nm was measured in the absorption spectra, and the corresponding fluorescence spectra also showed a visible decrease (~18%). Compared to the linkage of the aptamer, smaller *Δλ*_LSPR_ and a lower decline of fluorescence intensity occurred after capturing AFP. The reason is that, according to the evanescent field theory, the enhanced electric fields of Au NRs driving from LSPR decay rapidly with distance from the Au NRs surfaces [58,59]. The distance between the AFP protein and the Au NR-QDs apta-sensor is farther than the distance between the aptamer and Au NR-QDs, which lead to a lower perturbation of the electric field and are shown as a smaller spectral response. The TEM images of Au NR-QDs (a) and Au NR-QDs apta-sensor-AFP (b) were shown in Figure 3D, respectively. After negative-stained preparations, a transparent white layer (1.9 ± 0.3 nm) was observed on the surface of Au NR-QDs (b), which was assumed to be the AFP protein that was captured. The stability of the Au NR-QDs apta-sensor was also studied, and the fluorescence intensity of the Au NR-QDs apta-sensor was kept stable in 3 h (Figure S9). We successfully obtained an Au NR-QDs apta-sensor and it can be further applied for one-step detection of AFP.

### 3.4. Au NR-QDs Apta-sensor for AFP Detection

**Figure 4:**
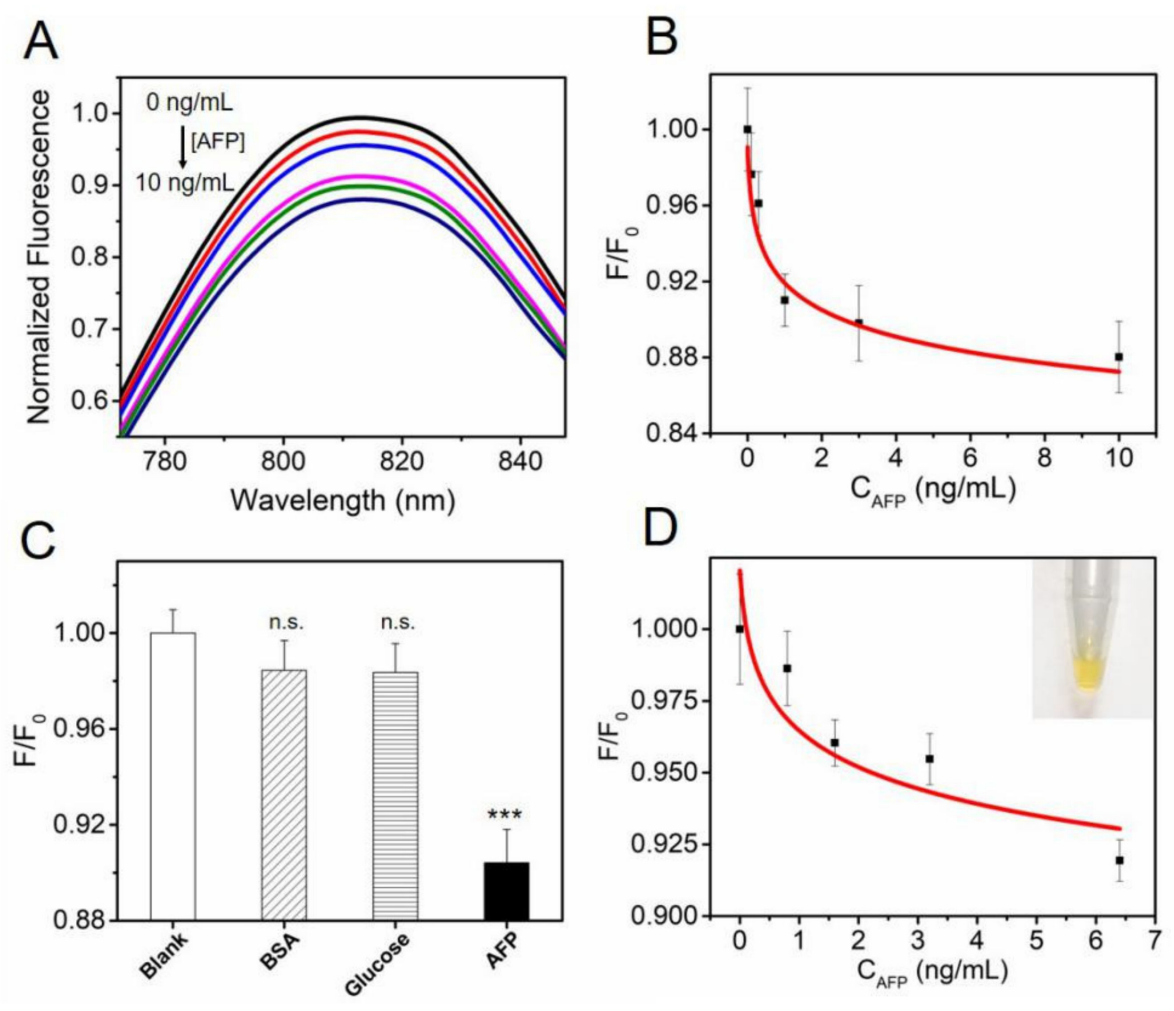
Sensitivity and selectivity of as-prepared Au NR-QDs apta-sensor for AFP detection. (**A**) Normalized fluorescence spectra of Au NR-QDs apta-sensor incubated with different concentrations of AFP from 0 to 10 ng/mL, where the maximum fluorescence intensity of Au NR-QDs apta-sensor was regarded as 1.0. (**B**) The relative fluorescence intensities (*F/F_0_*) against the AFP concentration exacted from (**A**). (**C**) The variation of *F/F_0_* for Au NR-QDs apta-sensor treated with PBS (blank), bull serum albumin (BSA, 1 μg/mL), glucose (100 ng/mL), and AFP (10 ng/mL), respectively. The asterisks indicated the significant differences between blank and experiment groups (*** *p* < 0.001, n.s. *p* > 0.05). (**D**) Detection of AFP in spiked human serum with as-prepared Au NR-QDs apta-sensor. The relative fluorescence intensities (*F/F*_0_) showed a similar negative relationship against AFP concentration in human serums. The inset is the picture of human serum.

The sensitivity of as-prepared Au NR-QDs apta-sensor for AFP detection was further investigated. Au NR-QDs apta-sensors were incubated with AFP from 0 to 10 ng/mL, and the corresponding fluorescence intensity clearly declined with the ascend of AFP concentration (Figure 4A). A power function dependence was also observed between *F/F*_0_ and the concentration of AFP (*F/F*_0_ = 0.920(*C_AFP_* + 0.040)^−0.023^, *R*^2^ = 0.93) in Figure 4B, which followed the similar change tendency as the RI response experiment in Figure 2. The 10 ng/mL of AFP was selected as the upper concentration when under incubation with Au NR-QDs apta-sensors because the changes of fluorescence intensity at AFP concentrations that was higher than 10 ng/mL were almost the same to the concentration of 10 ng/mL (unshown data). This indicates that the ability of Au NR-QDs apta-sensors to capture AFP may be saturated around the AFP concentration of 10 ng/mL. The limit of detection (LOD) of AFP was calculated as 0.30 ng/mL, which was far below the critical AFP concentration for early diagnoses of HCC (i.e., 25 ng/mL [11]) while an Au NRs apta-sensor was also constructed for AFP detection and showed almost no response to AFP from 0 to 50 ng/mL, which suggests that the sensing system of Au NRs based on *Δλ*_LSPR_ is not sensitive enough to detect AFP for early HCC diagnoses (Figure S10). Thus, our plasmon-regulated FRET sensing strategy can effectively improve the sensitivity of the plasmonic biosensor and realize the one-step detection of AFP. 

Furthermore, the selectivity of the Au NR-QDs apta-sensor for AFP detection is also evaluated by incubating Au NR-QDs apta-sensors with PBS, bull serum albumin (BSA), glucose, and AFP under the same condition, respectively. As shown in Figure 4C, the capture of AFP could cause a remarkable decrease of fluorescence intensity (*p* < 0.001), while the variations of fluorescence intensity were negligible (*p* > 0.05) in groups of these interfering agents with higher concentrations. The results confirm the sufficient sensitivity and selectivity of as-prepared Au NR-QDs apta-sensor for AFP detection.

We further applied an as-prepared Au NR-QDs apta-sensor to practical detection of AFP in spiked serum samples. Standard solutions of AFP with different concentrations were spiked into negative serums respectively and then incubated with Au NR-QDs apta-sensor (the final concentration is from 0 to 6.4 ng/mL, Figure 4D). The fluorescence intensity showed a similar change with the increase of AFP concentration as the detection of AFP in water (*F/F*_0_ = 0.966(*C_AFP_* + 0.064)^−0.020^, *R*^2^ = 0.80). The LOD for AFP detection in spiked serum sample was calculated to be 1.44 ng/mL, which effectively meets the need for clinical detection of AFP. Additionally, the concentrations of AFP in three spiked serum samples were determined, and the results were summarized in Table 1. Recoveries of known spiked amounts of AFP in three different serum samples were between 104.3% and 118% with the relative standard deviation (RSD) being lower than 10%, which suggests the potential applicability of the Au NR-QDs apta-sensor for clinical detection of AFP. 

### 3.5. Construction of Au NR-QDs and Au NP-QDs Nano-sensors for cTnI Detection

**Figure 5:**
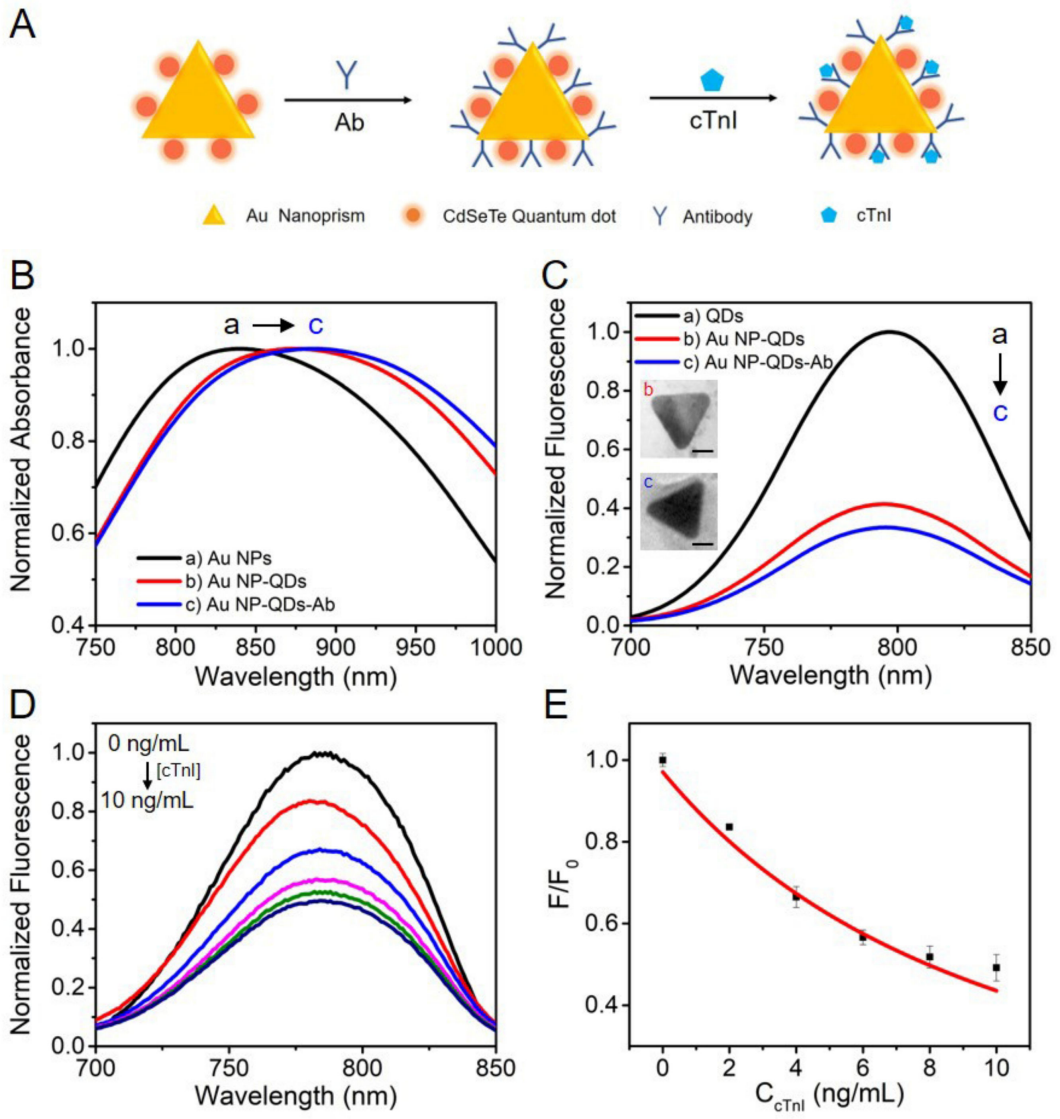
The antibodies-modified Au nano-prism-QDs (Au NP-QDs-Ab) nano-sensor for the detection of cardiac troponin I (cTnI). (**A**) Schematics for the construction of the Au NP-QDs-Ab nano-sensor and its application for cTnI detection. (**B**) Normalized UV-vis absorption spectra and (**C**) normalized fluorescence spectra during the fabrication process of the Au NP-QDs nano-sensor. The insets in (**C**) were the TEM images of Au NP-QDs (b) and Au NP-QDs-Ab (c). Scale bars are 20 nm. (**D**) Normalized fluorescence spectra of Au NP-QDs-Ab nano-sensor incubated with different concentrations of cTnI ranging from 0 to 10 ng/mL, where the maximum fluorescence intensity of Au NP-QDs sensor was regarded as 1.0. (**E**) The relationship of relative fluorescence intensities (*F/F*_0_) against cTnI concentration (*F/F_0_* = 131.3(*C_cTnI_* + 17.00)^−1.732^, *R*^2^ = 0.95) extracted from (**D**).

To demonstrate the universality of the plasmon-regulated FRET sensing strategy with random modification layers and plasmonic nanostructures, antibodies modified nano-sensors for cardiac troponin I (cTnI) detection were constructed based on Au NR-QDs and Au nano-prism-QDs (Au NP-QDs), respectively. An Au NR-QDs-Ab nano-sensor against cTnI was constructed as shown in Figure S11. A similar sensing performance was observed (*R*^2^ = 0.99) for the Au NR-QDs-Ab nano-sensor with a LOD of 0.75 ng/mL. Furthermore, with Au nano-prisms as plasmonic nanostructures, an Au NP-QDs-Ab nano-sensor was constructed as shown in Figure 5A. Antibodies can be also modified onto the surfaces of Au NPs by the crosslinking of –NH_2_. The absorption and fluorescence spectra for the fabrication process of Au NP-QDs-Ab nano-sensor is shown in Figure 5B,C. The linkage of QDs on the surfaces of Au NPs caused a ~34 nm redshift of *λ*_LSPR_ and a ~60% decrease of fluorescence intensity. After the modification of antibodies on the surfaces of Au NP-QDs, another ~11 nm redshift of *λ*_LSPR_ and ~20% decrease of fluorescence intensity were observed. Then, the sensitivity of as-prepared Au NP-QDs nano-sensor for cTnI detection was demonstrated. A remarkable decrease of fluorescence intensity occurred with the increasing concentration of cTnI (Figure 5D). There was also a power function type relationship between *F/F_0_* and cTnI concentration as we expected (*F/F*_0_ = 131.3(*C_cTnI_* + 17.00)^−1.732^, *R*^2^ = 0.95) with the LOD of 0.33 ng/mL, which was lower than the critical concentration of cTnI (2.0 ng/mL) in healthy human serum (Figure 5E) [60]. The results suggest that the universality of our plasmon-FERT sensing system enables the detection for different analytes by changing the modification layer and/or plasmonic nanostructures.

## 4. Conclusions

In summary, we have designed a plasmon-regulated FRET sensing system of Au NR-QDs with regulated *E_t_* for sensitive and selective detection of biomarkers. The regulated *E_t_* arises from the tunable LSPR properties of plasmonic nanostructures against the RI change. It was first reported that the absorption spectra of the plasmonic structure in a plasmon-emitter hybrid nanostructure were employed to regulate *E_t_* and further applied for analytes detection. This plasmon-regulated FRET sensing system possesses the following unique features: (1) good sensing reversibility, (2) effectively improved sensitivity, compared with the sensing performance of the plasmonic substrate based on the shift of LSPR, (3) the ability for serving as a universal sensing platform for the detection of various analytes due to the simple and flexible fabrication and modification process. Certainly, the present work was still confronted with some limitations. For example, the sensitivity for the detection of analytes in a wide concentration range is limited, and the anti-interference ability needs to be improved. Further studies will aim to increase the selectivity and try to realize the high throughput sensing of multiplex analytes by simultaneously introducing various fluorescent materials with different emission peaks into one plasmon-regulated FRET nano-sensor. In all, our proposed plasmon-regulated FRET strategy is encouraging to develop general sensing platforms with the advantages of easy fabrication and high sensitivity for high-throughput, real-time, label-free, and multi-analyte sensing applications in the field of chemical and biological analysis.

## Figures and Tables

**Table 1:** The application of gold nanorods-quantum dots (Au NR-QDs) apta-sensor for the determination of serum samples spiked with different concentrations of alpha fetoprotein (AFP).

Sample	Concentration of AFP Spiked (ng/mL)	Concentration of AFP Found (ng/mL)	Recovery (%)	RSD (%)
diluted serum 1	1.6	1.9	118	9.48
diluted serum 2	6.4	7.3	114	7.18
diluted serum 3	10	10.40	104	2.33

## Supplementary Materials

The following are available online at https://www.mdpi.com/2079-4991/10/3/444/s1. Figure S1: UV-vis spectra of Au NRs before and after the modification of cysteamine. Figure S2: Fabrication of Au NR-QDs assemblies using Au NRs with different LSPR peak wavelength. Figure S3: Fabrication of Au NR-QDs assemblies using QDs of different concentrations. Figure S4: The monitoring for the fabrication process of Au NR-QDs assemblies. Figure S5: TEM images of QDs and Au NRs. Table S1: Zeta-potentials of different structures during the fabrication of Au NR-QDs. Figure S6: The response of Au NRs to RI change. Figure S7: The fluorescence stability of QDs against RI. Figure S8: Sensing reversibility and stability of Au NR-QDs upon reversible changes of RI. Figure S9: The fluorescence stability of the Au NR-QDs apta-sensor. Figure S10: Au NRs apta-sensor for AFP detection from 0.1 to 50 ng/mL. Figure S11: Construction of an antibodies-modified Au NR-QDs sensor for cTnI detection.

## References

[1] Maccaferri N., Zhao Y.Q., Isoniemi T., Iarossi M., Parracino A., Strangi G., De Angelis F. (2019). Hyperbolic meta-antennas enable full control of scattering and absorption of light. Nano Lett..

[2] Shen Y., Zhou J.H., Liu T.R., Tao Y.T., Jiang R.B., Liu M.X., Xiao G.H., Zhu J.H., Zhou Z.K., Wang X.H. (2013). Plasmonic gold mushroom arrays with refractive index sensing figures of merit approaching the theoretical limit. Nat. Commun..

[3] Mayer K.M., Hafner J.H. (2011). Localized surface plasmon resonance sensors. Chem. Rev..

[4] Tang L.H., Li J.H. (2017). Plasmon-based colorimetric nanosensors for ultrasensitive molecular diagnostics. ACS Sens..

[5] Anker J.N., Hall W.P., Lyandres O., Shah N.C., Zhao J., Van Duyne R.P. (2008). Biosensing with plasmonic nanosensors. Nat. Mater..

[6] Sreekanth K.V., Alapan Y., ElKabbash M., Ilker E., Hinczewski M., Gurkan U.A., De Luca A., Strangi G. (2016). Extreme sensitivity biosensing platform based on hyperbolic metamaterials. Nat. Mater..

[7] Liu B.W., Chen S., Zhang J.C., Yao X., Zhong J.H., Lin H.X., Huang T.X., Yang Z.L., Zhu J.F., Liu S. (2018). A plasmonic sensor array with ultrahigh figures of merit and resonance linewidths down to 3 nm. Adv. Mater..

[8] Wang Y.Y., Zhou J.H., Li J.H. (2017). Construction of plasmonic nano-biosensor-based devices for point-of-care testing. Small Methods.

[9] Szunerits S., Boukherroub R. (2012). Sensing using localised surface plasmon resonance sensors. Chem. Commun..

[10] Culver H.R., Wechsler M.E., Peppas N.A. (2018). Label-free detection of tear biomarkers using hydrogel-coated gold nanoshells in a localized surface plasmon resonance-based biosensor. ACS Nano.

[11] Li W., Jiang X., Xue J., Zhou Z., Zhou J. (2015). Antibody modified gold nano-mushroom arrays for rapid detection of alpha-fetoprotein. Biosens. Bioelectron..

[12] Sabela M., Balme S., Bechelany M., Janot J.M., Bisetty K. (2017). A review of gold and silver nanoparticle-based colorimetric sensing assays. Adv. Eng. Mater..

[13] Coglitore D., Janot J.M., Balme S. (2019). Protein at liquid solid interfaces: Toward a new paradigm to change the approach to design hybrid protein/solid-state materials. Adv. Colloid Interface Sci..

[14] Li J.F., Li C.Y., Aroca R.F. (2017). Plasmon-enhanced fluorescence spectroscopy. Chem. Soc. Rev..

[15] Kim J.E., Choi J.H., Colas M., Kim D.H., Lee H. (2016). Gold-based hybrid nanomaterials for biosensing and molecular diagnostic applications. Biosens. Bioelectron..

[16] Wang M.S., Krasnok A., Zhang T.Y., Scarabelli L., Liu H., Wu Z.L., Liz-Marzan L.M., Terrones M., Alu A., Zheng Y.B. (2018). Tunable Fano resonance and plasmon-exciton coupling in single Au nanotriangles on monolayer WS_2_ at room temperature. Adv. Mater..

[17] Luk’yanchuk B., Zheludev N.I., Maier S.A., Halas N.J., Nordlander P., Giessen H., Chong C.T. (2010). The Fano resonance in plasmonic nanostructures and metamaterials. Nat. Mater..

[18] Ramirez M.O., Molina P., Gomez-Tornero A., Hernandez-Pinilla D., Sanchez-Garcia L., Carretero-Palacios S., Bausa L.E. (2019). Hybrid plasmonic-ferroelectric architectures for lasing and SHG processes at the nanoscale. Adv. Mater..

[19] Rakovich A., Albella P., Maier S.A. (2015). Plasmonic control of radiative properties of semiconductor quantum dots coupled to plasmonic ring cavities. ACS Nano.

[20] Werschler F., Lindner B., Hinz C., Conradt F., Gumbsheimer P., Behovits Y., Negele C., de Roo T., Tzang O., Mecking S. (2018). Efficient emission enhancement of single CdSe/CdS/PMMA quantum dots through controlled near-field coupling to plasmonic bullseye resonators. Nano Lett..

[21] Zhang X., Hu Y., Yang X., Tang Y., Han S., Kang A., Deng H., Chi Y., Zhu D., Lu Y. (2019). Forster resonance energy transfer (FRET)-based biosensors for biological applications. Biosens. Bioelectron..

[22] Ling J.A., Huang C.Z. (2010). Energy transfer with gold nanoparticles for analytical applications in the fields of biochemical and pharmaceutical sciences. Anal. Methods.

[23] Bitton O., Gupta S.N., Haran G. (2019). Quantum dot plasmonics: From weak to strong coupling. Nanophotonics.

[24] Lee J.H., Cho H.Y., Choi H.K., Lee J.Y., Choi J.W. (2018). Application of gold nanoparticle to plasmonic biosensors. Int. J. Mol. Sci..

[25] Jeong Y., Kook Y.M., Lee K., Koh W.G. (2018). Metal enhanced fluorescence (MEF) for biosensors: General approaches and a review of recent developments. Biosens. Bioelectron..

[26] Abadeer N.S., Brennan M.R., Wilson W.L., Murphy C.J. (2014). Distance and plasmon wavelength dependent fluorescence of molecules bound to silica-coated gold nanorods. ACS Nano.

[27] Aissaoui N., Moth Poulsen K., Kall M., Johansson P., Wilhelmsson L.M., Albinsson B. (2017). FRET enhancement close to gold nanoparticles positioned in DNA origami constructs. Nanoscale.

[28] Fang A.J., Chen H.Y., Li H.T., Liu M.L., Zhang Y.Y., Yao S.Z. (2017). Glutathione regulation-based dual-functional upconversion sensing-platform for acetylcholinesterase activity and cadmium ions. Biosens. Bioelectron..

[29] Xia Y., Song L., Zhu C. (2011). Turn-on and near-infrared fluorescent sensing for 2,4,6-trinitrotoluene based on hybrid (gold nanorod)-(quantum dots) assembly. Anal. Chem..

[30] Qu A.H., Xu L.G., Sun M.Z., Liu L.Q., Kuang H., Xu C.L. (2017). Photoactive hybrid AuNR-Pt@Ag_2_S core-satellite nanostructures for near-infrared quantitive cell imaging. Adv. Funct. Mater..

[31] He W., Sun X., Liu B., Shen J. (2019). A label-free “SEF-FRET” fluorescent sensing platform for ultrasensitive DNA detection based on AgNPs SAMs. Talanta.

[32] Zhou L., Ji F., Zhang T., Wang F., Li Y., Yu Z., Jin X., Ruan B. (2019). An fluorescent aptasensor for sensitive detection of tumor marker based on the FRET of a sandwich structured QDs-AFP-AuNPs. Talanta.

[33] Xu D.D., Liu C., Li C.Y., Song C.Y., Kang Y.F., Qi C.B., Lin Y., Pang D.W., Tang H.W. (2017). Dual amplification fluorescence assay for alpha fetal protein utilizing immunohybridization chain reaction and metal-enhanced fluorescence of carbon nanodots. ACS Appl. Mater. Interfaces.

[34] Kim E.J., Kim E.B., Lee S.W., Cheon S.A., Kim H.J., Lee J., Lee M.K., Ko S., Park T.J. (2017). An easy and sensitive sandwich assay for detection of Mycobacterium tuberculosis Ag85B antigen using quantum dots and gold nanorods. Biosens. Bioelectron..

[35] Yang S.H., Zhang F.F., Wang Z.H., Liang Q.L. (2018). A graphene oxide-based label-free electrochemical aptasensor for the detection of alpha-fetoprotein. Biosens. Bioelectron..

[36] Haran G., Chuntonov L. (2018). Artificial plasmonic molecules and their interaction with real molecules. Chem. Rev..

[37] Li M., Cushing S.K., Wang Q.Y., Shi X.D., Hornak L.A., Hong Z.L., Wu N.Q. (2011). Size-dependent energy transfer between CdSe/ZnS quantum dots and gold nanoparticles. J. Phys. Chem. Lett..

[38] Bujak L., Ishii T., Sharma D.K., Hirata S., Vacha M. (2017). Selective turn-on and modulation of resonant energy transfer in single plasmonic hybrid nanostructures. Nanoscale.

[39] Yu M.Q., Wang H., Fu F., Li L.Y., Li J., Li G., Song Y., Swihart M.T., Song E.Q. (2017). Dual-recognition Forster resonance energy transfer based platform for one-step sensitive detection of pathogenic bacteria using fluorescent vancomycin-gold nanoclusters and aptamer-gold nanoparticles. Anal. Chem..

[40] Huang C.J., Lin H.I., Shiesh S.C., Lee G.B. (2012). An integrated microfluidic system for rapid screening of alpha-fetoprotein-specific aptamers. Biosens. Bioelectron..

[41] Nikoobakht B., El Sayed M.A. (2003). Preparation and growth mechanism of gold nanorods (NRs) using seed-mediated growth method. Chem. Mater..

[42] Wang C., Irudayaraj J. (2008). Gold nanorod probes for the detection of multiple pathogens. Small.

[43] Maity A.R., Stepensky D. (2016). Efficient subcellular targeting to the cell nucleus of quantum dots densely decorated with a nuclear localization sequence peptide. ACS Appl. Mater. Interfaces.

[44] Shi Y., Zhang H., Yue Z., Zhang Z., Teng K.S., Li M.J., Yi C., Yang M. (2013). Coupling gold nanoparticles to silica nanoparticles through disulfide bonds for glutathione detection. Nanotechnology.

[45] Wang C., Qian J., Wang K., Yang X., Liu Q., Hao N., Wang C., Dong X., Huang X. (2016). Colorimetric aptasensing of ochratoxin A using Au@Fe_3_O_4_ nanoparticles as signal indicator and magnetic separator. Biosens. Bioelectron..

[46] Wu H.Y., Huang W.L., Huang M.H. (2007). Direct high-yield synthesis of high aspect ratio gold nanorods. Cryst. Growth Des..

[47] Haldar K.K., Sen T., Patra A. (2010). Metal conjugated semiconductor hybrid nanoparticle-based fluorescence resonance energy transfer. J. Phys. Chem. C.

[48] Stanisavljevic M., Krizkova S., Vaculovicova M., Kizek R., Adam V. (2015). Quantum dots-fluorescence resonance energy transfer-based nanosensors and their application. Biosens. Bioelectron..

[49] Gaur G., Koktysh D.S., Weiss S.M. (2013). Immobilization of quantum dots in nanostructured porous silicon films: Characterizations and signal amplification for dual-mode optical biosensing. Adv. Funct. Mater..

[50] Link S., Mohamed M.B., El-Sayed M.A. (1999). Simulation of the optical absorption spectra of gold nanorods as a function of their aspect ratio and the effect of the medium dielectric constant. J. Phys. Chem. B.

[51] Cui M., Wang Y., Jiao M., Jayachandran S., Wu Y., Fan X., Luo X. (2017). Mixed self-assembled aptamer and newly designed zwitterionic peptide as antifouling biosensing interface for electrochemical detection of alpha-fetoprotein. ACS Sens..

[52] Breaker R.R. (2004). Natural and engineered nucleic acids as tools to explore biology. Nature.

[53] Song S.P., Wang L.H., Li J., Zhao J.L., Fan C.H. (2008). Aptamer-based biosensors. Trends Anal. Chem..

[54] Wang W.T., Wang W., Davis J.J., Luo X.L. (2015). Ultrasensitive and selective voltammetric aptasensor for dopamine based on a conducting polymer nanocomposite doped with graphene oxide. Microchim. Acta.

[55] Coglitore D., Giamblanco N., Kizalaite A., Coulon P.E., Charlot B., Janot J.M., Balme S. (2018). Unexpected hard protein behavior of BSA on gold nanoparticle caused by resveratrol. Langmuir.

[56] Zhang Y.L., Qi S.J., Liu Z.G., Shi Y.P., Yue W.Q., Yi C.Q. (2016). Rapid determination of dopamine in human plasma using a gold nanoparticle-based dual-mode sensing system. Mater. Sci. Eng. C.

[57] Hu W.W., Chen Q.S., Li H.H., Ouyang Q., Zhao J.W. (2016). Fabricating a novel label-free aptasensor for acetamiprid by fluorescence resonance energy transfer between NH_2_-NaYF_4_: Yb, Ho@SiO_2_ and Au nanoparticles. Biosens. Bioelectron..

[58] Mock J.J., Hill R.T., Tsai Y.J., Chilkoti A., Smith D.R. (2012). Probing dynamically tunable localized surface plasmon resonances of film-coupled nanoparticles by evanescent wave excitation. Nano Lett..

[59] Liu Z., Lee H., Xiong Y., Sun C., Zhang X. (2007). Far-field optical hyperlens magnifying sub-diffraction-limited objects. Science.

[60] Han X., Li S.H., Peng Z.L., Othman A.M., Leblanc R. (2016). Recent development of cardiac troponin I detection. ACS Sens..

