# Supplementary material for: Plasmon-Emitter Hybrid Nanostructures of Gold Nanorod-Quantum Dots with Regulated Energy Transfer as a Universal Nano-Sensor for One-Step Biomarker Detection
Source: Nanomaterials (Basel). 2020 Mar 1;10(3):444. doi: 10.3390/nano10030444 (PMC7152990; doi:10.3390/nano10030444)
Supplement: Supplementary file 1 [file nanomaterials-10-00444-s001.pdf]

Supplementary Materials for:

Plasmon-Emitter Hybrid Nanostructures of Gold Nanorod-Quantum Dots with Regulated Energy Transfer as a Universal Nano-Sensor for One-step Biomarker Detection

Xuemeng Li [1,†], Yingshuting Wang [1,†], Quanying Fu [1], Yangyang Wang [1], Dongxu Ma [1], Bin Zhou [1] and Jianhua Zhou [1,2,*]

1 Key Laboratory of Sensing Technology and Biomedical Instruments of Guangdong Province, School of Biomedical Engineering, Sun Yat-sen University, Guangzhou 510275, China; lixuemeng6758@126.com (X.L.); wangysht@mail2.sysu.edu.cn (Y.W.); fuqy3@mail2.sysu.edu.cn (Q.F.); wang45yangyang@163.com (Y.W.); ma023ma@126.com (D.M.); zhoub56@mail2.sysu.edu.cn (B.Z.)

2 Division of Engineering in Medicine, Department of Medicine, Brigham and Women's Hospital, Harvard Medical School, Cambridge, MA 02139, USA

* Correspondence: zhoujh33@mail.sysu.edu.cn; Tel./Fax: +86-20-3938-7890

† The authors contributed equally to this work.

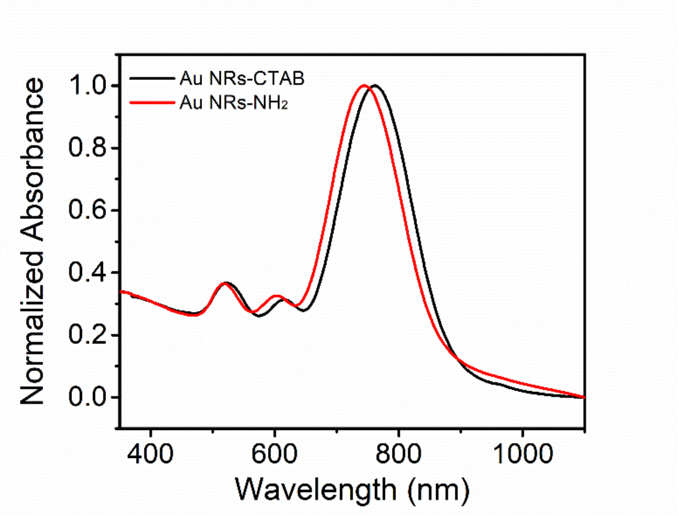

Figure S1. UV-vis spectra of Au NRs before and after the modification of cysteamine. A blueshift of LSPR peak was observed due to the decrease of RI around Au NRs, since the cysteamine molecule is smaller than the bilayer of CTAB molecule.

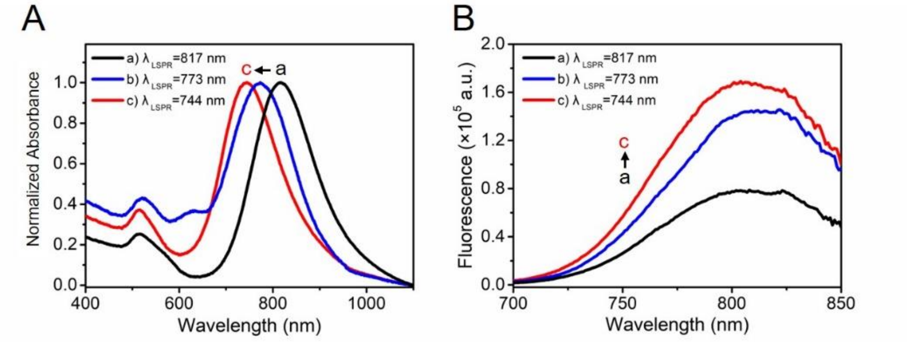

Figure S**2**. Fabrication of Au NR-QDs assemblies using Au NRs with different LSPR peak wavelength (λ_{LSPR}). (A) Normalized UV-vis absorption spectra of Au NR-QDs using Au NRs with different λ_{LSPR}: λ_{LSPR}=817 nm (a), 773 nm (b) and 744 nm (c), respectively. (B) Corresponding fluorescence spectra of Au NR-QDs during the same process in (A). The fluorescence intensity of Au NR-QDs increased with the λ_{LSPR} of Au NRs decreasing from 817 nm to 744 nm.

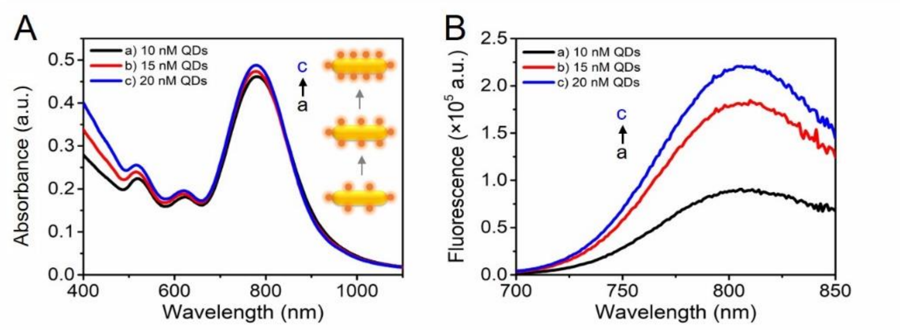

Figure S3. Fabrication of Au NR-QDs assemblies using QDs with different concentrations. (A) UV-vis absorption spectra of Au NR-QDs by using QDs of 10, 15 and 20 nM reacted with Au NRs of 0.115 nM, respectively. The molar ratio of Au NRs: QDs were calculated as 1:90, 1: 130 and 1:170, respectively. (B) Corresponding fluorescence spectra of Au NR-QDs during the same process in (A).

The concentration of Au NRs can be calculated by the Lambert-Beer law:

$$A = \varepsilon bc \tag{1}$$

where A is the absorbance of Au NRs for a given wavelength, ε is the corresponding extinction coefficient, b is the thickness of absorbent substance, and c is the concentration of the Au NRs. According to a previous study [1], the molar extinction coefficient of Au NRs with peak wavelength at 744 nm (ε) is 4.14×10^9 $M^{-1}cm^{-1}$. When the absorbance of Au NRs at 744 nm (A) is 0.48 and the thickness of absorbent substance (b) is 1 cm, the concentration of Au NRs (c) was calculated to be 0.115 nM.

Au NRs of 0.115 nM was used to react with different concentrations of QDs (10, 15 and 20 nM) in which the molar ratio of Au NRs: QDs were about 1:90, 1: 130 and 1:170, respectively. To obtain relatively high fluorescence intensity and appropriative low consumption of CdSeTe QDs, we chose QDs of 15 nM reacting with Au NRs of 0.115 nM in the following experiments

(molar ratio of Au NRs: QDs was about 1:130).

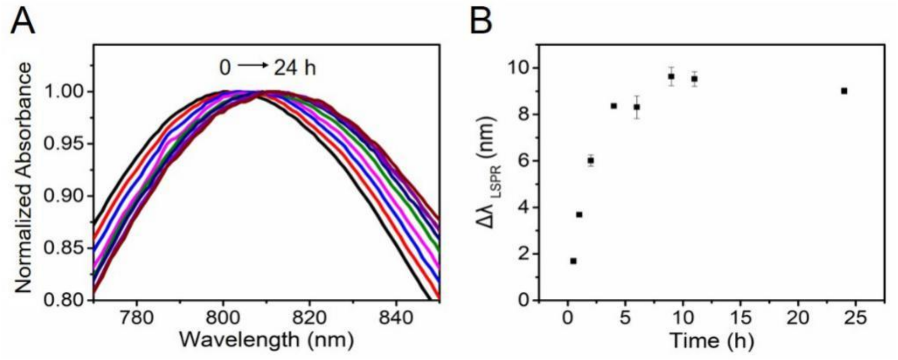

Figure S4. The monitoring for the fabrication process of Au NR-QDs assemblies. (A) Normalized UV-vis absorption spectra of Au NR-QDs at different incubation time (t=0, 0.5, 1, 2, 4, 6, 9, 11 and 24 h). (B) The relationship between the shift of λ_{LSPR} ($\Delta\lambda_{LSPR}$) and different incubation time extracted from (A). The fabrication process of Au NR-QDs was completed after 9 h incubation.

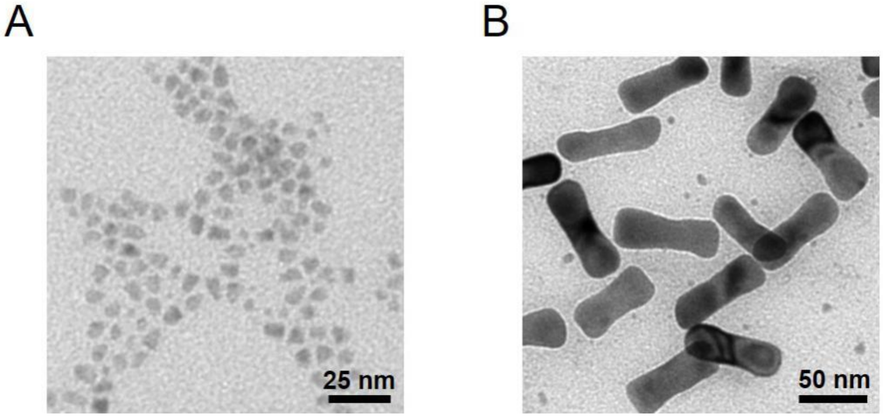

Figure S5. TEM images of (A) QDs and (B) Au NRs, respectively. The size of Au NRs for ~20 particles on the basis of the TEM micrographs have been calculated with an average length of 54±2 nm and an average diameter of 16±1 nm.

A series of experiments were conducted to ascertain the optimal conditions for the fabrication of Au NR-QDs: (1) Au NRs with absorption peak (λ_{LSPR}) of 744 nm were chosen, since the effective fluorescence quenching of QDs with Au NRs was observed in comparison with the original fluorescence of pure QDs, and the relatively high fluorescence intensity of Au NR-QDs was beneficial to record the fluorescence quenching in the follow-up experiments (Figure S2). (2) The molar ratio of Au NRs: QDs=1:130 was selected to prepare Au NR-QDs with relatively high fluorescence intensity and appropriately low consumption of QDs (Figure S3). (3) The assembling process of Au NR-QDs was completed after the 9 h of incubation (Figure S4). TEM images of QDs and Au NRs were presented in Figure S5. In brief, Au NR-QDs assemblies were fabricated by covalently linking QDs (emission peak of 812 nm) with Au

NRs (λ_{LSPR} of 744 nm), and the molar ratio of Au NRs: QDs was 1:130 for the follow-up experiments.

Table S1. Zeta-potentials of different structures during the fabrication of Au NR-QDs.

Materials	Zeta (mV)
Au NRs-CTAB	35.3±0.6
Au NRs-NH_2	21.2±6.8
QDs	-16.2±3.1
Au NR-QDs	-23.7±1.4

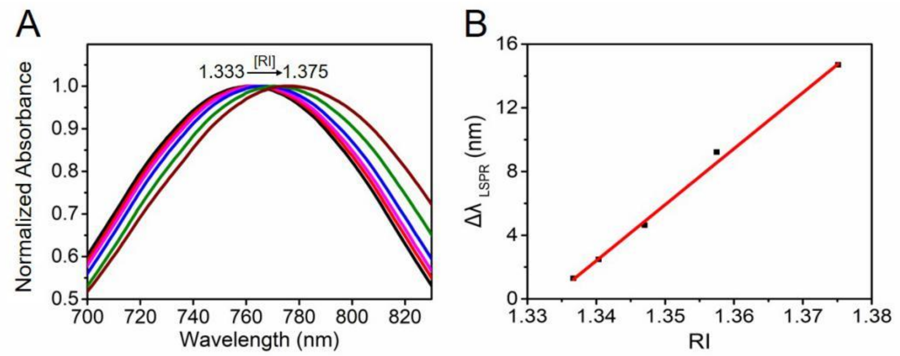

Figure S6. The response of Au NRs to refractive index (RI) change. (A) Normalized UV-vis absorption spectra of Au NRs in various RI conditions (n=1.333, 1.337, 1.340, 1.344,1.347, 1.350, 1.357 and 1.375). (B) Relationship between the $\Delta\lambda_{LSPR}$ and RI, extracted from (A). The $\Delta\lambda_{LSPR}$ showed good linearity with RI (R^2=0.99), and the slope indicated that the RI sensitivity of Au NRs was ~363 nm/RIU. Each measurement was repeated three times, and the error bars are too small to be seen.

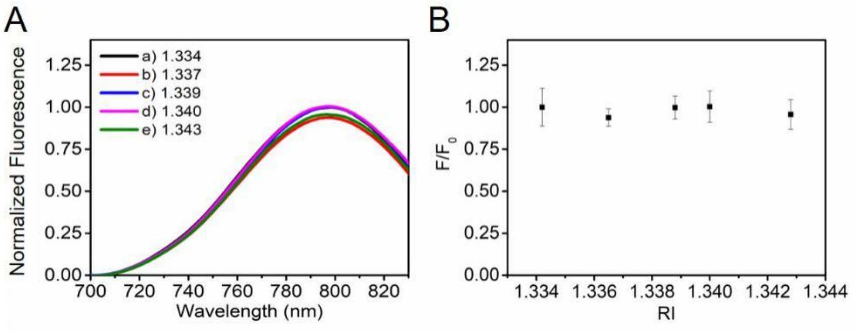

Figure S7. The fluorescence stability of QDs against RI. (A) Normalized fluorescence spectra of QDs in different RI conditions (n=1.334, 1.337, 1.339, 1.340 and 1.343). (B) The relationship between relative fluorescence intensity (F/F_0) and RI, in which the fluorescence intensity of QDs at 812 nm with RI value of 1.334 was defined as F_0. The fluorescence intensity of pure QDs was relatively stable in varied RI.

Calculations of theoretical F/F_0 (F'/F'_0) based on the experimental absorption spectra of Au NR-QDs

In a fluorescence resonance energy transfer (FRET) system, the energy transfer efficiency is defined as eq. 2 [2]:

$$E = \frac{{R_0}^6}{{R_0}^6 + R^6} \tag{2}$$

where R_0 is the separation distance of fluorescence donor and acceptor at which the energy transfer efficiency E is 50%, and R is the distance between the fluorescence donor and acceptor.

R_0 can be described as eq. 3 [3]:

$$R_0 = 0.211[\kappa^2 n^{-4} \phi_D S_{OL}]^{1/6} \tag{3}$$

where κ^2 is the dipole orientation factor which varies from 0 to 4 and describes the average squared orientational part of a dipole-dipole interaction, n is the refractive index (RI) of the medium, Φ_D designates the quantum yield of the donor, and S_{OL} expresses the spectral overlap integral between the normalized donor emission and the acceptor absorption.

Besides, the energy transfer efficiency E can be also evaluated by eq. 4 [4]:

$$E = 1 - \frac{F_{AD}}{F_D} \tag{4}$$

where F_{AD} and F_D are the donor emission in the presence and absence of acceptor, respectively.

So, F_{AD}/F_D can be simplified to the following:

$$\frac{F_{AD}}{F_D} = \frac{1}{Bn^{-4}S_{OL} + 1} \tag{5}$$

with

$$\mathrm{B} = \frac{8.82 \times 10^{-5}\kappa^2 \phi_D}{R^6} \tag{6}$$

where factor B is a constant that contains the quantum yield of the donor Φ_D, dipole orientation factor κ^2, and the interparticle distance between fluorescence donor and acceptor R.

For a FRET system which holds a large level of energy transfer efficiency, eq. 5 can be simplified to the eq. 7:

$$\frac{F_{AD}}{F_D} = \frac{1}{Bn^{-4}S_{OL}} \tag{7}$$

So, when the change of RI is applied to influence the fluorescence intensity of a FRET system, the relative fluorescence intensity (F/F_0) can be calculated according to the eq. 7:

$$\frac{F}{F_0} = \frac{n^4}{AS_{OL}} \tag{8}$$

with

$$\mathrm{A} = \frac{{\mathrm{n}_0}^4}{\mathrm{S}_{\mathrm{OL0}}} \tag{9}$$

where F is the fluorescence intensity of the FRET system under different RI conditions, F_0 is F in an initial RI environment as reference, n_0 is the surrounding RI of F_0, and S_{OL0} is the corresponding spectral overlap integral of F_0.

In the case of our Au NR-QDs system, the values of the overlap integrals between normalized absorption spectra and normalized fluorescence spectra are S_{OL}=82.481, 82.991,

83.103, 83.208, 83.304, 83.385, 83.483 and 83.524 for various RI values (n=1.333, 1.336, 1.337, 1.339, 1.340, 1.341, 1.343 and 1.344), respectively. S_{OL} increased when the RI changed from 1.333 to 1.344, and the increase of it tended to slow down. We chose a power function to fit it: S_{OL}= $85.83\Delta n^{0.006}$ (R^2 = 0.99) with Δn = n-1.332.

Then, n_0=1.333, S_{OL0}=82.48 and S_{OL}=$85.83\Delta n^{0.006}$ were substituted into eq. 8, so the theoretical F/F_0 (F'/F'_0) can be calculated as follows:

$$\frac{F'}{F'_0} = \frac{1}{0.038} \times \frac{(\Delta n + 1.332)^4}{85.83\Delta n^{0.006}} \quad (10)$$

In our system, the value of n is within the range of 1.333 ≤ n ≤ 1.344. So, terms in eq. 10 containing the element of Δn can be neglected since the values of them are far less than the fourth power of 1.332 (3.148).

Thus, the relationship between theoretical F'/F'_0 and n can be approximately equal to the following:

$$\frac{F'}{F'_0} = 0.965\Delta n^{-0.006} = 0.965(n - 1.332)^{-0.006} \quad (11)$$

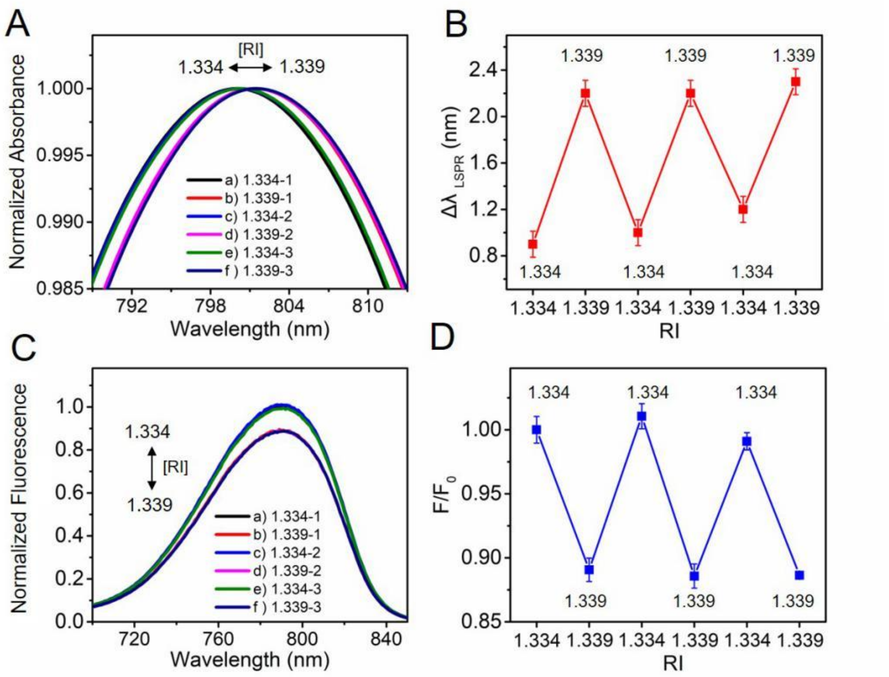

Figure S8. Sensing reversibility of Au NR-QDs upon reversible changes of RI. (A) Normalized UV-vis absorption spectra of Au NR-QDs between RI values of 1.334 and 1.339 for three cycles. (B) The $\Delta\lambda_{LSPR}$ of Au NR-QDs upon the variation of RI were extracted from (A). (C) Normalized fluorescence spectra of Au NR-QDs in the same process of (A). (D) Relative fluorescence intensities (F/F_0) at 812 nm upon the variation of RI were extracted from (C), in which the fluorescence intensity of Au NR-QDs at 812 nm with RI value of 1.334 in the first measurement was defined as F_0. The Au NR-QDs presented good sensing reversibility and stability in the reversible RI processes between 1.333 and 1.339.

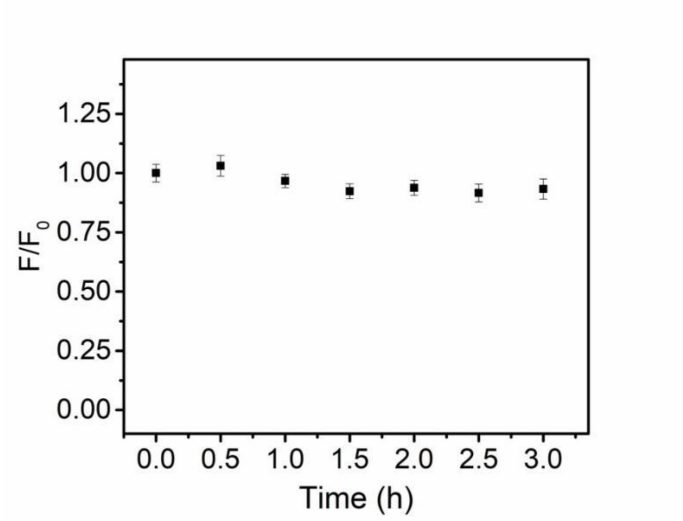

Figure S9. The fluorescence stability of Au NR-QDs aptasensor. Fluorescence intensity of the Au NR-QDs aptasensor kept stable in 3 hours.

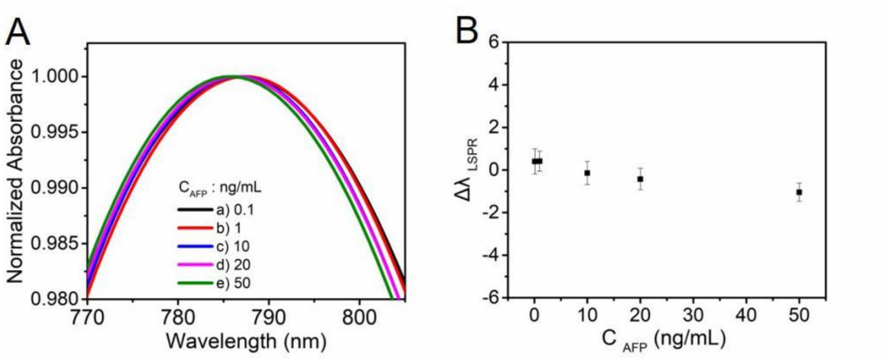

Figure S10. Au NRs aptasensor for AFP detection from 0.1 to 50 ng/mL. (A) Normalized UV-vis absorption spectra of Au NRs aptasensor in various concentrations of AFP. (B) The $\Delta\lambda_{LSPR}$ against AFP concentration. The $\Delta\lambda_{LSPR}$ showed almost no response to AFP, suggesting that the Au NRs aptasensor is not sensitive enough to detect AFP in the concentration range from 0.1 to 50 ng/mL.

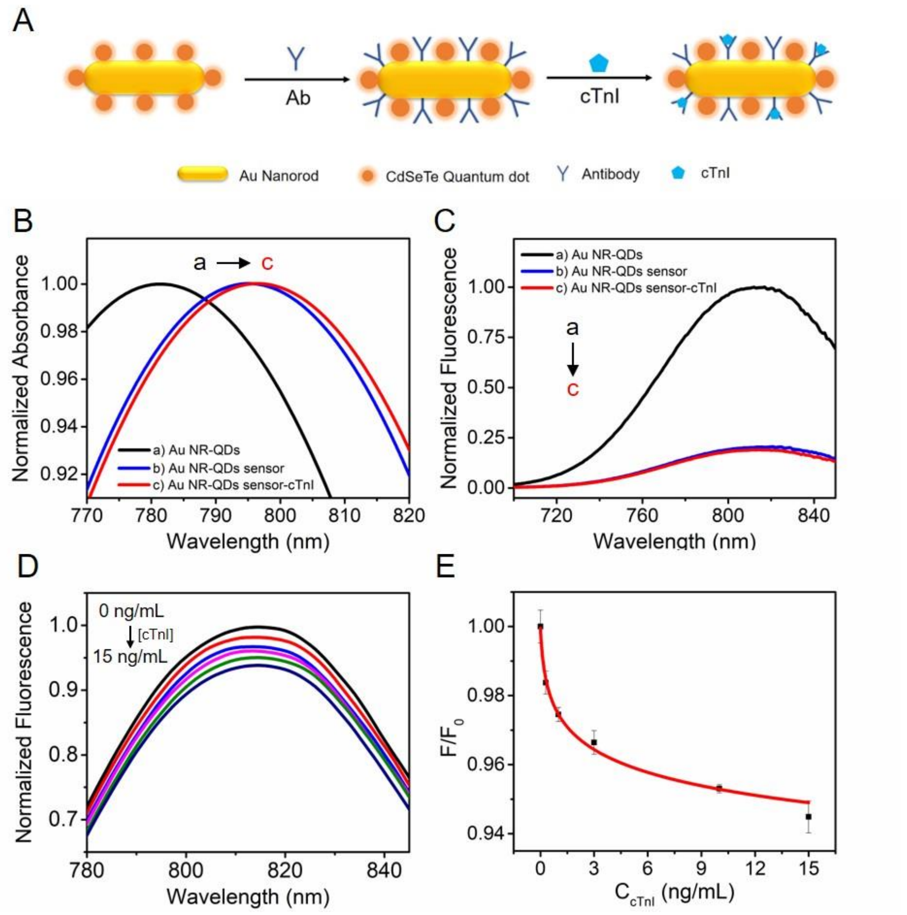

Figure S11. Construction of an antibodies-modified Au NR-QDs sensor for cTnI detection. (A) Schematics for the construction of the antibody-modified Au NR-QDs nanosensors and its application for cTnI detection. (B) Normalized UV-vis absorption spectra and (C) normalized fluorescence spectra of Au NR-QDs (a), Au NR-QDs sensor (b) and Au NR-QDs sensor-cTnI nanocomplex (c). The λ_{LSPR} visibly red-shifted and the fluorescence intensities decreased after each operation. (D) Normalized fluorescence spectra of Au NR-QDs sensor incubated with different concentrations of cTnI from 0 to 15 ng/mL. (E) The relationship of relative fluorescence intensities (F/F_0) against cTnI concentration (F/F_0= 0.976(C_{AFP}+0.091)$^{-0.010}$, R^2=0.99) that exacted form (A).

Reference

1. Orendorff, C. J.; Murphy, C. J., Quantitation of metal content in the silver-assisted growth of gold nanorods. *J. Phys. Chem. B* **2006,** *110*, 3990-3994.
2. Lakowicz, J. R., *Principles of fluorescence spectroscopy*. 3rd ed.; Springer: Boston, 2006.
3. Qiao, Y.; Polzer, F.; Kirmse, H.; Steeg, E.; Kuhn, S.; Friede, S.; Kirstein, S.; Rabe, J. P., Nanotubular J-aggregates and quantum dots coupled for efficient resonance excitation energy transfer. *ACS Nano* **2015,** *9*, 1552-1560.
4. Gordon, G. W.; Berry, G.; Xiao, H. L.; Levine, B.; Herman, B., Quantitative fluorescence resonance energy transfer measurements using fluorescence microscopy. *Biophys. J.* **1998,** *74*, 2702-2713.
